# An unexpected diversity of trypanosomatids in fecal samples of great apes

**DOI:** 10.1016/j.ijppaw.2018.09.003

**Published:** 2018-09-05

**Authors:** Jan Votýpka, Barbora Pafčo, David Modrý, Donald Mbohli, Nikki Tagg, Klára J. Petrželková

**Affiliations:** a Department of Parasitology, Faculty of Science, Charles University, Prague, Czech Republic; b Institute of Parasitology, Czech Academy of Sciences, České Budějovice, Czech Republic; c Department of Pathology and Parasitology, Faculty of Veterinary Medicine, University of Veterinary and Pharmaceutical Sciences, Brno, Czech Republic; d Central European Institute for Technology (CEITEC), University of Veterinary and Pharmaceutical Sciences, Brno, Czech Republic; e Association de la Protection de Grands Singes, Centre for Research and Conservation, Royal Zoological Society of Antwerp, Antwerp, Belgium; f Institute of Vertebrate Biology, Czech Academy of Sciences, Brno, Czech Republic; g Liberec Zoo, Liberec, Czech Republic

**Keywords:** *Leishmania*, *Herpetomonas*, Trypanosomatids, Detection, Gorilla, Chimpanzee, Feces, PCR

## Abstract

Charismatic great apes have been used widely and effectively as flagship species in conservation campaigns for decades. These iconic representatives of their ecosystems could also play a role as reservoirs of several zoonotic diseases. Recently it was demonstrated that African great apes can host *Leishmania* parasites (Kinetoplastea: Trypanosomatidae). Given that this finding raised a strong negative reaction from leishmania experts and the subsequent discussion did not lead to a clear resolution, we decided to analyze wild gorilla (*Gorilla gorilla gorilla*) and chimpanzee (*Pan troglodytes troglodytes*) fecal samples collected from the same area in Cameroon as in the original study. Fecal samples, used to circumvent the difficulties and ethics involved in obtaining blood samples from endangered wild apes, were screened by three different PCR assays for detection of *Leishmania* DNA. We did not detect any leishmania parasites in analyzed feces; however, sequencing of SSU rRNA revealed an unexpected diversity of free-living bodonids (Kinetoplastea: Bodonidae) and parasitic trypanosomatids (Kinetoplastea: Trypanosomatidae) other than *Leishmania*. A single detected *Phytomonas* species, found in chimpanzee feces, most likely originated from animal plant food. On the other hand, the presence of four free-living bodonid species and four parasitic insect monoxenous trypanosomatid, including two possible new species of the genus *Herpetomonas*, could be explained as *ex post* contamination of feces either from the environment or from flies (Diptera: Brachycera).

## Highlights

• We analyzed wild gorilla and chimpanzee fecal samples, from the area where a previous study detected *Leishmania* parasites.
• We detected leishmania DNA neither in great ape fecal samples nor in feces of experimentally infected rodents.
• We revealed unexpected diversity of bodonids, monoxenous and dixenous trypanosomatids other than *Leishmania.*

## Graphical abstract

**Figure:**
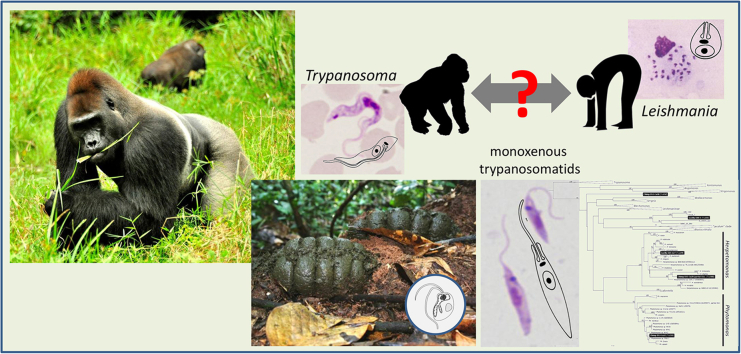

## 1 Introduction

Great apes, by their very nature, are extremely vulnerable. Today, these highly intelligent, charismatic species are threatened by many factors, including destruction of their forest habitats, hunting for bushmeat and the illegal pet trade, and disease. Circulation of pathogens between free ranging primates and local human population attract a lot of attention, as primates might be reservoirs for several diseases of man and vice versa.

Due to the relatively recent and surprising findings that gorillas could play a role in the transmission of human parasitic diseases, such as malaria (e.g., Prugnolle et al., 2011) and strongylid nematodes (Hasegawa et al., 2014), and because of the newly recorded observation of trypanosomes in wild chimpanzee feces (Jirků et al., 2015), it is important to explore the potential presence of protozoan parasites in great apes. In 2015, Hamad et al. (2015a) reported *Leishmania major* parasites in feces of wild western lowland gorillas originating from southern Cameroon. The detection was based on PCR and fluorescence in situ hybridization. This highly unexpected finding elicited a negative reaction by a group of experts with extensive experience of working in the field of leishmaniasis research (Bastien et al., 2015). The comments of Bastien et al. (2015) were published together with a reply from the authors of the original contribution (Hamad et al., 2015b).

Through this discussion, both groups of authors put forward a number of arguments supporting their claims and refuting the counterclaims. Bastien et al. (2015) raised arguments based mainly on well-known epidemiological facts, argued about the promastigote morphotype detected in the gorilla feces, and discussed the methods used. However, despite the long-term experience of both author groups in their respective scientific fields, the information provided in the communication (original article, comments and reply) is not sufficient to allow us to conclude whether or not gorillas are a possible reservoir of *Leishmania major*, and if leishmania parasites (namely promastigotes and amastigotes) are detectable in the feces of great apes. The original findings of Hamad et al. (2015a) are interesting and turn our attention to the importance of the role of great apes in the transmission, circulation and spreading of human diseases. Recent studies (Hamad et al., 2015a; Jirků et al., 2015) suggest the involvement of great apes in the circulation of dixenous trypanosomatids; in particular, those of the genera *Trypanosoma* and *Leishmania* (belonging to the same family Trypanosomatidae). However, there is no evidence that leishmaniasis and/or trypanosomiasis are present in the resident human population living in the study area (Hamad et al., 2015a; Bastien et al., 2015).

We believe that the findings of Hamad et al. (2015a) require further investigation. We therefore decided to contribute to this stimulating discussion by analyzing newly-acquired gorilla and chimpanzee fecal samples from an area adjacent to the origin of the samples analyzed by Hamad et al. (2015a). The newly-collected samples were analyzed using well-established methodology for various trypanosomatid and blood parasite detection that is commonly used in our laboratories to investigate mainly trypanosomes, leishmanias and plasmodium (e.g., Myšková et al., 2008; Jirků et al., 2015; Mapua et al., 2016; Ionică et al., 2017). From a large number of methods regularly used for *Leishmania* detection (see Akhoundi et al., 2017), we chose four, based on qPCR, nested PCR and conventional PCR and targeting three different DNA loci.

## 2 Materials and methods

Fecal samples of free-ranging central chimpanzees (*Pan t. troglodytes*; N = 25) and western lowland gorillas (*Gorilla g. gorilla*; N = 50) were collected from September to October 2014 at the northern periphery of the Dja Faunal Reserve in Cameroon around the research site La Belgique with approval of the Ministry of Scientific Research and Innovation and the Ministry of Forests and Wildlife, Cameroon. Feces were collected noninvasively from within gorilla night nests and from the forest floor under chimpanzee night nests, early in the morning (i.e., before 9am), thus ensuring that we collected only fresh feces. Samples were extracted from within the core of the feces and placed in collection tubes containing RNAlater. Samples were kept at room temperature for a maximum of one month before transport to the laboratory, where they were subsequently stored at −70 °C until further processing. DNA isolation was performed from approximately 3g of fecal homogenate using the QIAamp DNA Stool Mini Kit. A stool sample from a human volunteer was used as a negative DNA control; and two positive controls were prepared from two human volunteer stool samples each mixed with approximately 100 cells of cultured *Trypanosoma ranarum* or *Leishmania tarentolae* and kept for one week in RNAlater at −70 °C before DNA isolation.

Four different polymerase chain reaction (PCR) assays were accomplished according to previously-established protocols. While quantitative PCR (qPCR) used kinetoplast DNA (kDNA) as the target (Myšková et al., 2008), conventional PCR protocols (Schönian et al., 2003) and nested approach (Jirků et al., 2015) was used to amplify a region of the Internal Transcribed Spacer 1 (ITS-1). Both these targets, kDNA and ITS-1, were used only for leishmania and/or trypanosome detection. On the contrary, less specific nested PCR protocol for SSU rRNA (Seward et al., 2017) amplify all members of the order Kinetoplastea. Positive samples on nested SSU rRNA PCR were subjected to subsequent direct sequencing and phylogenetic analysis as described by Schoener et al. (2018). The final dataset contained 314 taxa and 3339 positions.

Each sample was independently subjected to each of the aforementioned PCRs at least twice, and more often three times. The results of the above mentioned different PCR protocols have always been consistent, as reported elsewhere (e.g., Akhoundi et al., 2017).

In order to test whether it is possible to detect leishmania and/or trypanosome parasites in feces, we infected five BALB/c mice by *Trypanosoma brucei brucei*, four mice by a cutaneous strain of *Leishmania major*, and three hamsters by a viscerotropic strain of *L. infantum*. The fecal pellets were collected weekly up to two month after infection and analyzed by above-mentioned PCR protocols.

## 3 Results

No parasite DNA belonging to the genera *Trypanosoma* or *Leishmania* was detected in any of the analyzed samples by any of the used PCR assays. On the other hand, we detected using nested PCR targeting SSU rRNA parasitic trypanosomatids other than *Leishmania*/*Trypanosoma,* as well as free-living bodonids (both being members of the order Kinetoplastea), in the great ape fecal samples. Five gorilla samples contained three species of bodonids, *Bodo* aff. *caudatus* (D17, D19-66, D59), *Parabodo nitrophilus* (D50) and *Neobodo* aff. *designis* (D67), and two chimpanzee samples were found positive for two bodonid species, *Neobodo* aff. *designis* (D55-Cu34) and *Neobodo* sp. 1 (D08-Cu11).

**Fig. 1:**
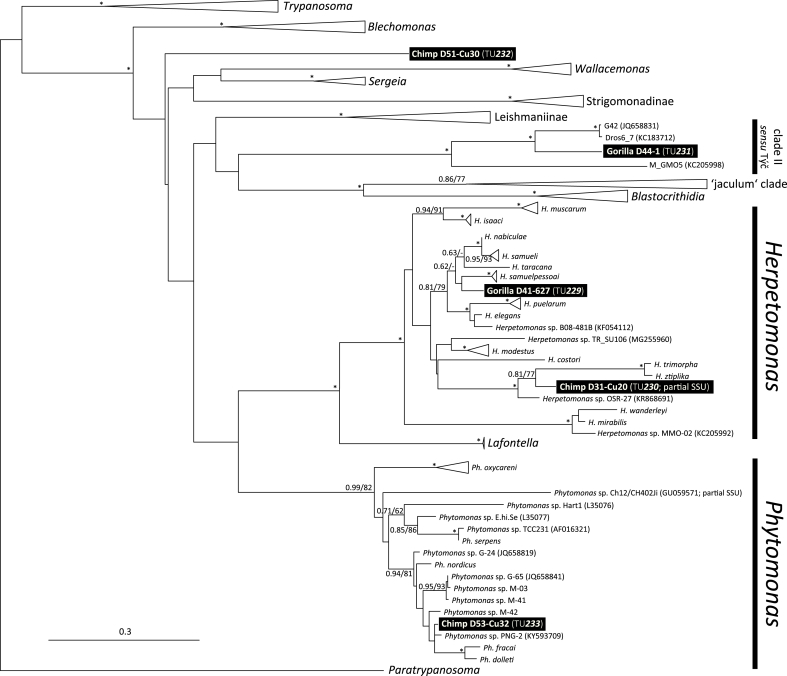
**Phylogenetic relationships of the detected trypanosomatids.** An SSU rRNA-based Bayesian phylogenetic tree of trypanosomatid sequences (∼2 kb) obtained from gorilla and chimpanzee fecal samples collected in the Dja Faunal Reserve in Cameroon representing the most likely two new *Herpetomonas* species, one unknown *Phytomonas* species and two most likely new monoxenous trypanosomatid species of unnamed genera. These possible new species are assigned as new Typing Units (TUs) with numbers TU***229***–***233***. Bootstrap values from Bayesian posterior probabilities (MrBayes; 5 million generations) and bootstrap percentages for maximum-likelihood analysis (PhyML; 1000 replicates) are shown at the nodes; dashes indicate <50% bootstrap support or different topology; asterisks mark branches with maximal statistical support. The tree was rooted with *Paratrypanosoma*; the closest relative of the family Trypanosomatidae. Parasite names or names of strains supplemented with their GenBank accession numbers are given; the branch lengths are drawn proportionally to the amount of changes (scale bar).

Furthermore, we found several representatives of monoxenous trypanosomatids, mostly members of the genus *Herpetomonas*. Of four *Herpetomonas*-positive fecal samples (two of each studied great ape species), sufficiently long (∼2000 bp) SSU rRNA sequences were obtained from one gorilla sample (D41-627; GenBank accession number is MG845925) and one chimpanzee sample (D31-Cu20; MG845928) and the subsequent phylogenetic analysis revealed two possible new herpetomonad species designated as new typing units, TU***299*** and TU***300*** (see Fig. 1). A nucleotide sequence-based approach allows substitution of a species that lacks detailed morphological descriptions with operational proxies – typing units (TUs) delineated in terms of sequence divergence of the appropriate marker (Maslov et al., 2013; Lukeš et al., 2018).

Another fecal sample (Gorilla, D44-1; MG845924) contained a parasite DNA which represents a possible new species (TU***231***) within the as yet unnamed cosmopolitan genus of monoxenous trypanosomatids (provisionally referred to as clade-II; *sensu* Týč et al., 2013) with a high affinity to Diptera fly hosts (J.V. and V. Yurchenko, unpubl. data). We also detected an SSU rRNA sequence in a chimpanzee sample (D51-Cu30a; MG845926) which represents a new species (TU***232***), an entirely new lineage (and thus a possible new genus) (see Fig. 1) within the family Trypanosomatidae. Finally, we found a possible new *Phytomonas* species (TU***233***; see Fig. 1) in three chimpanzee samples (D11-Cu14, D13-Cu16 and D53-Cu32; MG845926).

Experimental infection of rodents succeeded in all cases. *Trypanosoma brucei brucei* develops heavy infection in blood with serious symptoms and experimental mice had to be killed seven days after infection. Mice infected by *Leishmania major* developed skin ulcerating lesions on injected ears, while *L. infantum* parasites developed in the spleen and liver and caused only moderate symptoms in infected hamsters. While the trypanosome infections were detectable in the feces of three out of five infected mice using the above-mentioned PCR protocols, we did not detect leishmania DNA in any fecal sample of the experimentally infected rodents.

## 4 Discussion

The primary motivation of our study was to verify the previous findings and confirm/refute the occurrence of *Leishmania* spp. in gorillas and chimpanzees by detecting of parasite DNA in great ape feces coming from the same area as in the original study (Hamad et al., 2015a). In previous studies, we have used various detection methodologies for thousands of samples including fecal samples, and the results arising from our research have appeared in numerous studies (e.g., Myšková et al., 2008; Jirků et al., 2012, 2015; Týč et al., 2013; Votýpka et al., 2015a; Mapua and Votýpka, 2015; Mapua et al., 2015, 2016; Ionică et al., 2017). Due to the prevalence of previous rigorously acquired results, the established and long-term nature of our work in this scientific field, and our experience in the detection of leishmania parasites (see Akhoundi et al., 2017), we feel competent to have conducted such a study.

No *Trypanosoma*/*Leishmania* DNA was detected using four different PCR assays; on the other hand, we revealed unexpected diversity of free-living bodonids and parasitic trypanosomatids in great ape feces. The absence of trypanosome or leishmania parasites in the studied great ape feces is in accordance with the epidemiological situation of the study area where, to the best of our knowledge, neither human leishmaniasis nor trypanosomiasis has been reported. In addition to this, we did not detect leishmania DNA in any fecal sample of the experimentally infected rodents. Our result suggests that it may not be possible to detect leishmania parasites in fecal samples; however, we are aware that the situation in the rodent lab models may not correspond to that of natural reservoirs. Nery et al. (2015) report the occurrence of *L. infantum* amastigotes and DNA in dog feces; however, canine visceral leishmaniasis develops in hosts' inner organs, has a serious effect on infected symptomatic dogs and parasite occurrence in their feces is therefore expected. Unlike this visceral form, cutaneous leishmaniasis is different, restricted to the host skin and, therefore, regardless of whether the infection is symptomatic or asymptomatic, we would not expect to be able to detect the parasites in mice or great ape feces.

We have detected four bodonid species, *Bodo* aff. *caudatus*, *Parabodo nitrophilus*, *Neobodo* aff. *designis*, and *Neobodo* sp. 1, in studied great ape feces. Although naturally free-living aquatic bodonids, the sister clade of parasitic trypanosomatids, have been very rarely identified in vertebrate hosts (for a review see Szőke et al., 2017), we believe that, in the present study, the occurrence of bodonids may be the result of secondary contamination of feces from the external environment or that the isolated DNA originated from bodonids present in water consumed by the apes.

In the last decades, it has been demonstrated that monoxenous trypanosomatids are very common parasites or commensals of insects and the number of newly described species or typing units (TUs) is constantly increasing (see Maslov et al., 2013; Votýpka et al., 2015b; Lukeš et al., 2018). Entirely in agreement with this statement, we reveal four new monoxenous trypanosomatid TUs in our study. There are two possible explanations of the origin of the two *Herpetomonas* spp. and the two unnamed species detected in the ape feces. Fecal samples could have been contaminated with feces of flies (Diptera), which are the principal hosts of these parasites. Flies use feces as the main food source for their larvae and can penetrate ape fecal boules within minutes after deposition. The feces were collected from unhabituated apes, and therefore not immediately after defecation (see Materials and methods), thus resulting in a strong likelihood of contamination by fly feces containing *Herpetomonas* parasites. The second potential explanation is that the apes consumed food items, for example fruit, that was previously contaminated by feces of flies infected with herpetomonads.

The dixenous genus *Phytomonas* is transmitted by a phytophagous insect vector (Heteroptera) and infects a variety of plant species. Because of its life-cycle, contamination of the feces from the surrounding environment is not probable, and thus the detected parasites are more likely to have passed through the chimpanzee digestive system; we therefore assume that the parasites found in the feces originated from chimpanzee food items. This is different to the above-mentioned case of free-living bodonids and monoxenous insect trypanosomatids in great ape feces, whereby it is highly likely that they were contaminated by items from the external environment (e.g., water, flies, etc.). Though it cannot be confirmed, we believe that the detection of phytomonad DNA in the analyzed great ape feces could demonstrate that the methods employed are suitable for the detection of dixenous trypanosomatid parasites (including leishmania) in this type of material.

To summarize, we found an unexpected diversity of free-living bodonids, insect monoxenous trypanosomatids and a member of the genus *Phytomonas* in the fecal samples of great apes from the northern periphery of the Dja Faunal Reserve. However, these microorganisms most likely enter the feces via contamination from the external environment or by passive passage through the digestive tract of the host. Since we detect leishmania DNA neither in great ape fecal samples nor in feces of experimentally infected rodents, we are unable to support the findings of Hamad et al. (2015a). We suggest that more ape samples from different sites across the Dja Faunal Reserve and its periphery and from other sites across Africa should be examined to clarify if great apes might play a role as reservoir hosts for *L. major* parasites.

## Conflicts of interest

All authors: No reported conflicts.

## Appendix A Supplementary data

Data Profile Data Profile
